# The Impact of the Digital Age and Social Media on Connecting the Clubfoot Community

**DOI:** 10.7759/cureus.16780

**Published:** 2021-07-31

**Authors:** Natalie Tonkovich, Danika Baskar, Steve Frick

**Affiliations:** 1 Orthopaedics, Stanford University, Stanford, USA

**Keywords:** clubfoot, talipes equinovarus, ponseti, social media, support, information sharing, community

## Abstract

Background

Internet chat rooms played an important role in the late 1990s promoting the Ponseti method as the preferred initial treatment for congenital clubfoot. The social media boom has created multiple new methods for caregivers to seek support from a global community using a variety of platforms that are now easily available. This study assesses the reach of information shared across social media platforms about congenital clubfoot and analyzes topics most commonly discussed among members in these groups.

Methodology

Posts and pages across Facebook, Instagram, Twitter, and TikTok were evaluated to identify the top clubfoot-related hashtags and accounts. In addition, content themes were analyzed for posts across all platforms.

Results

There were 122 clubfoot-focused Facebook groups for parent support, and the five Facebook groups with the highest number of posts during the study period were found to frequently discuss the following topics: successful treatment stories, questions about casting, bracing, relapse, and commercial items compatible with clubfoot treatment. Twitter pages contained information about live webinars, educational resources for parents and providers, and the impact of the coronavirus disease 2019 pandemic on clubfoot treatment. A search across visual platforms using “#clubfoot” yielded over 59,000 cumulative posts on Instagram and over 34.7 million total views on TikTok.

Conclusions

Parents of clubfoot patients are increasingly connecting through digital social media platforms, relying on them for information on clubfoot, and utilizing them as a network for social support. Clubfoot physicians should be aware of this content on social media to promote education and discussion that addresses parent concerns, provides accurate information, and guides expectations.

## Introduction

Ignacio Ponseti, M.D. developed a method for the treatment of congenital clubfoot at the University of Iowa in the late 1940s [1]. This method consisted of serial manipulations and plaster casts usually with tendo-Achilles tenotomy to achieve initial correction of the deformity, followed by splinting to maintain proper foot positioning. The goal was to avoid joint release surgeries, and he published excellent results of his method in the Journal of Bone and Joint Surgery (JBJS) in 1963 [2]. Further refinements of his method, including management of clubfoot relapse, were published over the ensuing years in a 1980 JBJS publication detailing excellent correction sustained with longer follow-up [3]. Despite Dr. Ponseti’s results and publication of evidence supporting his method, surgical treatment of clubfeet using a joint releasing approach to acutely realign the tarsal bones continued to remain the global standard of treatment for almost another two decades.

The rise in awareness of improved outcomes of the Ponseti method compared to joint-releasing surgery in the late 1990s coincided with three events: (1) the publication by Cooper and Dietz in 1995 of excellent results in a cohort of Dr. Ponseti’s clubfoot patients with a 30-year follow-up [4], (2) the publication of Dr. Ponseti’s book on fundamentals of treating congenital clubfoot in 1996 [1], and (3) the influence of the internet for parents and surgeons to learn about the Ponseti method [5]. Morcuende documented the important influence of the internet on parental decision-making for healthcare issues and specifically detailed how growing internet use by parents from 1998 to 2001 led many to learn about the Ponseti method from online sources and seek this method of treatment for their children rather than traditional surgical approaches [5].

Parents created clubfoot-focused internet support groups to provide information, share opinions, and provide support and encouragement to other families. One such notable Yahoo chat group called “NoSurgery4Clubfoot” was one of the earliest in which parents shared information about clubfoot treatment and the Ponseti method. In these chat rooms, if a parent was planning for traditional surgery, the group would recommend taking the child to Dr. Ponseti in Iowa for evaluation and treatment (personal communication, M. Egbert). Morcuende noted a substantial increase in clubfoot patients seeking treatment at the University of Iowa and attributed some of this growth to internet referrals [5]. As parents shared stories of successful treatment with the Ponseti method online, this mode of treatment grew in popularity and began gaining acceptance among the clubfoot provider community [5].

Since then, the use of the internet has increased exponentially and the advent of the social media boom has greatly expanded information-sharing capabilities across all aspects, including medical treatments and disease [6-8]. Shabtai et al. have documented the worldwide spread of the Ponseti method, and its acceptance as the “gold standard” for the treatment of congenital clubfoot, with information shared online playing a major role in its global adoption [9]. Today, parents continue to use the internet and social media platforms to gain disease-specific information about clubfoot, share information with other parents of clubfoot patients, and offer opinions and advice about caring for children with this condition [10-15]. This study examines the current use of social media by parents of children with clubfoot and presents common topics of discussion across various platforms from information gathered through a review of shared posts and interviews conducted with parent leaders of clubfoot support groups.

## Materials and methods

A search of posts across social media platforms including Facebook, Instagram, Twitter, and TikTok was conducted to identify pages with a primary focus on congenital clubfoot. Facebook was queried using the search terms “clubfoot,” “talipes,” and “equinovarus” to identify groups that were further stratified based on the following criteria: the type of group, total number of members, frequency of posts per month, the date the group was created, and the geographic location. Five groups that had the highest number of posts from September 1st, 2020 to October 19th, 2020 were further evaluated to identify the most commonly discussed topics. For each of these Facebook groups, all posts for the month of September 2020 were analyzed and categorized by content themes which were then compared between the groups.

A similar search was performed on Instagram, Twitter, and TikTok on April 15th, 2021 using the term “clubfoot,” from which the top five hashtags with the highest number of posts and views, along with the first five search results for social media pages were further categorized by content. Interviews were additionally conducted with parents of clubfoot children who are organizers and leaders of clubfoot-focused social media groups to assess the perceived value of parental participation in social media and to characterize the information sought and shared across these platforms.

## Results

**Figure 1:**
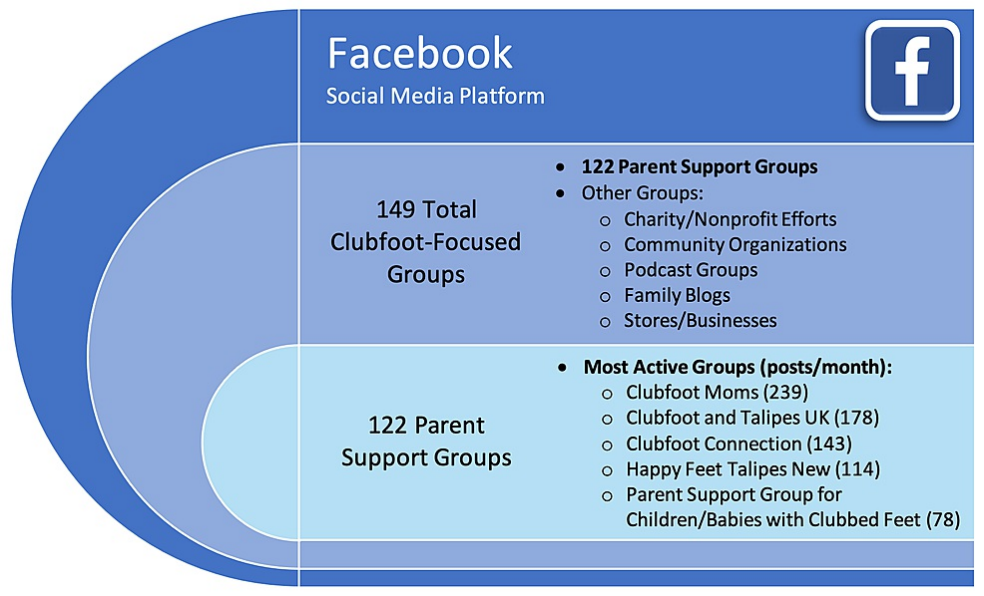
Analysis of identified clubfoot-focused Facebook groups.

**Figure 2:**
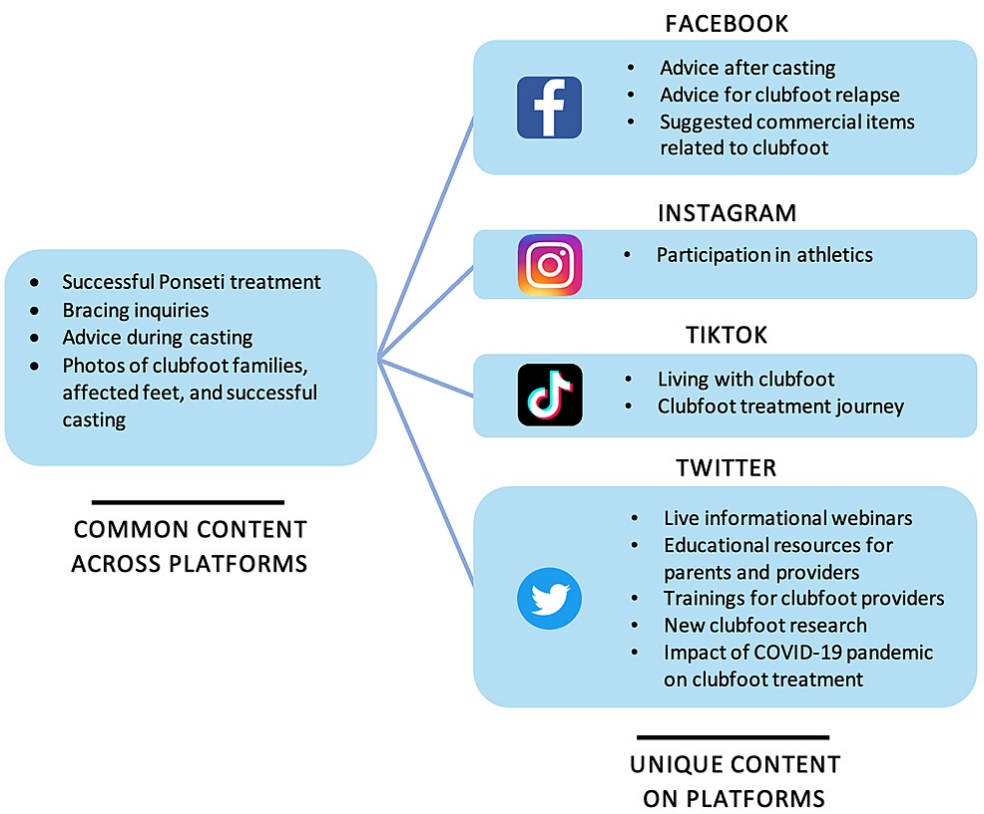
Content categories of shared posts across social media platforms.

A total of 149 Facebook clubfoot groups were identified, of which 122 were focused on providing a forum for parent discussion and support. The top five groups based on the highest number of posts per month were Clubfoot Moms, Clubfoot and Talipes UK, Clubfoot Connection, Happy Feet Talipes New, and Parent Support Group for Children/Babies With Clubfoot (Figure 1). The five most common topics of posts shared on Facebook were updates on the success of the Ponseti method of treatment, requests for advice regarding casting, questions about bracing, inquiries about clubfoot relapse, and suggested commercial items related to clubfoot (Figure 2).

**Figure 3:**
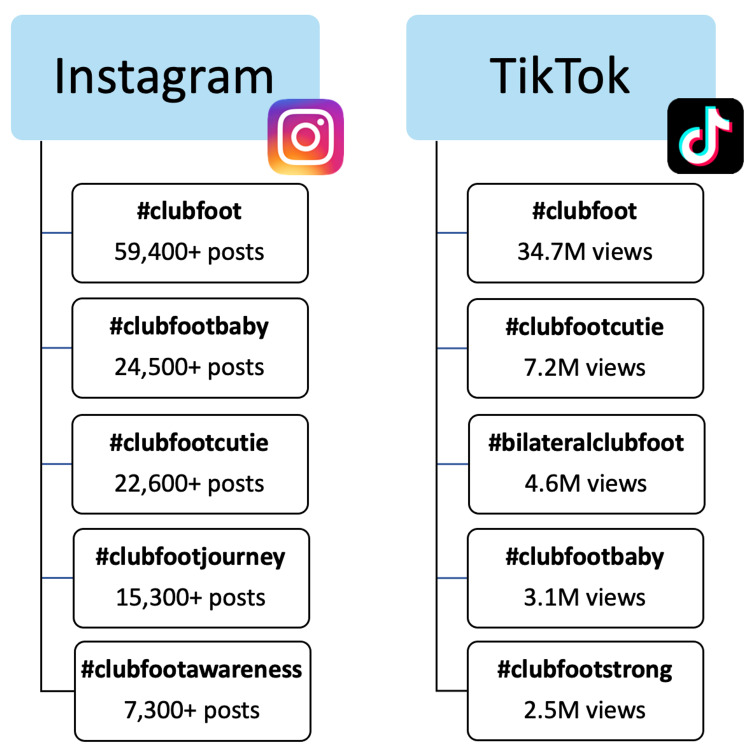
Instagram and TikTok hashtags with the highest number of clubfoot-related posts.

The first five search results for pages listed under “#clubfoot” on Instagram included @iowabrace, @clubfootmommas_, @clubfootcares, @clubfoot_warriors, and @clubfootjourney, with many of these pages having a following of over 1,000 members (Table 1). Clubfoot-related hashtags used to group posts on Instagram and TikTok are listed in Figure 3, with “#clubfoot” accumulating over 59,000 posts, and both “#clubfootbaby” and “#clubfootcutie” gathering over 22,000 posts each on Instagram. Content shared on TikTok using “#clubfoot” was found to have over 34.7 million cumulative views, with the other top hashtags also amounting to over 2 million total views each (Figure 3). The categories of content shared on Instagram and TikTok were focused on successful treatment stories, participation in athletics, photos and videos of babies with and without casts and braces, photos of mothers with their babies, and photos of affected clubfeet (Figure 2).

**Table 1:** Instagram and Twitter pages with the highest following.

Instagram page	Followers	Twitter page	Followers
@iowabrace	1,520	@miraclefeet	4,395
@clubfootmommas_	1,364	@STEPS_SA	1,898
@clubfootcares	1,288	@GlobalClubfoot	1,412
@clubfoot_warriors	325	@clubfootindia	648
@clubfootjourney	367	@IowaBrace	646

The Twitter social media platform was found to offer more educational and professional advice, led by @miraclefeet with more than 4,000 followers, followed by @STEPS_SA and @GlobalClubfoot, as shown in Table 1. Recent Twitter post subject matter included live webinars, educational resources, training for providers, sharing new clubfoot research, the impact of the coronavirus disease 2019 pandemic on ongoing work, and treatment success stories (Figure 2).

From interviews conducted with Martin Egbert and Jill Harold, two parents of children with clubfoot who are also actively involved with oversight for online parent support groups, the role of social media in guiding parents throughout their child’s clubfoot treatment journey was further explored from their unique perspective. In the late 1990s and early 2000s, parents of children with clubfoot began connecting on internet chat rooms to share treatment advice and experiences with each other. Parents who received successful casting treatment by Dr. Ponseti at the University of Iowa would comment in these groups encouraging others to consider this option, especially if surgery was being recommended as the primary form of treatment. Early on, most internet traffic was primarily in English, and as parents began traveling from other parts of the world to seek treatment in Iowa, knowledge of the Ponseti method started to expand as these families translated resources to their own languages and shared narratives of their experience among their respective regions (personal communication, M. Egbert). The information shared by parents on these online groups contains practical advice from parents who have experience caring for a child with clubfoot. Given the range of topics being discussed among parents in these groups, it is also not uncommon to find discussions about medical topics like casting, surgery, and opinions about treatment and providers which can present a concern as parents typically are speaking from personal experience. Overall, online clubfoot communities have increasingly connected families on a regional and international level to support them through their child’s treatment (personal communication, J. Harold).

## Discussion

The substantial role of the internet in spreading knowledge of the success of the Ponseti method for the treatment of congenital clubfoot in the United States and around the world has been recounted in previous literature [5,9]. A primary motive for parents to seek additional treatment options for clubfoot in the 1990s was to avoid traditional joint-invasive treatment with surgery. Despite 50 years of experience and publication of his works in major orthopedic journals, Ponseti and Morcuende from the University of Iowa credited involvement of parents and the growing influence of the internet as key drivers for the acceptance of the Ponseti method as the best initial clubfoot treatment. This study analyzed four major social media platforms including Facebook, Instagram, Twitter, and TikTok for information shared about clubfoot. Many groups were dedicated to supporting parents throughout various stages of treatment and providing clubfoot education and information. The main drivers for parents on social media are to provide emotional support, as well as medical and practical information about having a child with clubfoot undergo the Ponseti method of treatment. Organizations are using social media for education, fundraising, and sharing stories of successful treatment to promote awareness about the Ponseti method.

One of the major differences between early internet use, in contrast to today’s social media posting abilities, is the ease with which parents can share visual content, including photos or videos of their children’s feet, and request advice from hundreds or thousands of members on a public forum. This introduces concerns about the quality and medical validity of the assessments made and advice given based on photographic information alone. Another point to consider is that the majority of social media participants are parents of children with clubfoot who may be providing advice from personal experiences, and not physicians or Ponseti method practitioners. We found very few physicians participating in the identified social media groups. While physician participation could provide expertise and medical validation of shared content, offering medical opinions or advice in response to postings and photos on social media platforms without a full history and physical examination creates concerns about practicing medicine or telemedicine without proper licensing, credentialing, thorough evaluation, or consent. This can pose potential risks to members of social media support groups when well-intentioned support may cause more harm than good for a small percentage of users. Especially concerning is judging the treatment that has been provided thus far by other practitioners based on the limited information a post and/or photographs can provide. Thus, social media sites may provide medically incorrect advice when parents are speaking from experience about casting, diagnoses, surgery, and opinions about treatment without the scientific knowledge, physical examination findings, and clubfoot practice experience that the physician possesses.

In addition, some parents offer advice directing others to seek care with the Ponseti method practitioners they trust and continue to seek treatment from for their child. Similar to “word-of-mouth” referrals, this advice can be reassuring to parents with a clubfoot child diagnosed by prenatal ultrasound, or a newborn with clubfoot. Parents often seek reassurance about whether their child is receiving treatment according to Ponseti’s principles, about casts slipping, or the possibility that a complex clubfoot is developing [16]. While medical information about the Ponseti method can be delivered succinctly and clearly early on in the child’s treatment by having a conversation about the need for weekly casts, likely tendo-Achilles tenotomy, and full-time foot abduction bracing for three months followed by nighttime bracing for three to five years, parents of children with clubfoot have many more questions that are often best answered by other parents who have undergone similar experiences, emphasizing the value of social media support groups. During an interview with one of the leaders of a Facebook support group, she noted that the “group has lots of advice that even orthopaedic surgeons can never give you, but it comes from the experience of having a child with clubfoot” (J. Harold, personal communication). An example she shared was specific for parents who find out about a clubfoot diagnosis in utero. She mentioned these parents have a lot of time to blame themselves and may even think they did something wrong, but social media support groups can provide emotional support and an outlet for therapeutic discussion between those who share these common experiences.

As clubfoot is a birth defect that requires significant commitment from the child’s family for several years of early childhood for appropriate treatment, this makes the role of physicians in providing accurate information and setting realistic expectations for families even more imperative. For example, many parents may believe the Ponseti method can result in a “cure” for clubfoot, when in reality the axiom “once a clubfoot, always a clubfoot” holds true. Also, as the Ponseti method offers a simple, effective treatment that typically produces normal foot appearance and excellent function in childhood and into adulthood, many feet can still be challenging to treat, with up to 50% of patients experiencing relapse necessitating further treatment, and 22% of patients having fair or poor functional outcomes in adulthood [3]. Currently, few physicians were noted to participate in clubfoot social media sites, but increasing online physician presence and participation in these social media groups could help assure the accuracy of posted information and addressing misconceptions about treatment through education.

## Conclusions

Social media support groups play an increasingly important role in providing parents of children born with clubfoot with information about caring for them and continuing to promote the Ponseti method for the initial treatment of clubfoot over surgical intervention. Though not always the case with medical advice found on social media, the Ponseti method of treatment for clubfoot aligns with the prevailing view of the medical and scientific community. The ability of the internet and now social media platforms to connect parents of children with uncommon or rare disorders allows for the creation of a supportive community through which parents and their children may connect with other families facing similar health challenges. Physicians caring for clubfoot patients should be aware of the influence of social media on parental decision-making and may want to become involved in these platforms frequented by patient families. Healthcare organizations may also consider using social media to grow their online presence and ultimately expand accurate awareness and education about clubfoot and its treatment.
